# Properties of permutation-based gene tests and controlling type 1 error using a summary statistic based gene test

**DOI:** 10.1186/1471-2156-14-108

**Published:** 2013-11-07

**Authors:** David M Swanson, Deborah Blacker, Taofik AlChawa, Kerstin U Ludwig, Elisabeth Mangold, Christoph Lange

**Affiliations:** 1 Department of Biostatistics, Harvard School of Public Health, Boston, Massachusetts; 2 Departments of Psychiatry and Epidemiology, Massachusetts General Hospital, Boston, Massachusetts; 3 Institute of Human Genetics, University of Bonn, Bonn, Germany; 4 Department of Genomics, Life and Brain Center, University of Bonn, Bonn, Germany; 5 Institute for Genomic Mathematics; German Center for Neurodegenerative Diseases, University of Bonn, Bonn, Germany

**Keywords:** Dimension reduction, Eigenvector, Gene-based testing, Permutation tests

## Abstract

**Background:**

The advent of genome-wide association studies has led to many novel disease-SNP associations, opening the door to focused study on their biological underpinnings. Because of the importance of analyzing these associations, numerous statistical methods have been devoted to them. However, fewer methods have attempted to associate entire genes or genomic regions with outcomes, which is potentially more useful knowledge from a biological perspective and those methods currently implemented are often permutation-based.

**Results:**

One property of some permutation-based tests is that their power varies as a function of whether significant markers are in regions of linkage disequilibrium (LD) or not, which we show from a theoretical perspective. We therefore develop two methods for quantifying the degree of association between a genomic region and outcome, both of whose power does not vary as a function of LD structure. One method uses dimension reduction to “filter” redundant information when significant LD exists in the region, while the other, called the summary-statistic test, controls for LD by scaling marker Z-statistics using knowledge of the correlation matrix of markers. An advantage of this latter test is that it does not require the original data, but only their Z-statistics from univariate regressions and an estimate of the correlation structure of markers, and we show how to modify the test to protect the type 1 error rate when the correlation structure of markers is misspecified. We apply these methods to sequence data of oral cleft and compare our results to previously proposed gene tests, in particular permutation-based ones. We evaluate the versatility of the modification of the summary-statistic test since the specification of correlation structure between markers can be inaccurate.

**Conclusion:**

We find a significant association in the sequence data between the 8q24 region and oral cleft using our dimension reduction approach and a borderline significant association using the summary-statistic based approach. We also implement the summary-statistic test using Z-statistics from an already-published GWAS of Chronic Obstructive Pulmonary Disorder (COPD) and correlation structure obtained from HapMap. We experiment with the modification of this test because the correlation structure is assumed imperfectly known.

## Background

The focus in genetic association studies has been on uncovering loci that are risk factors for an outcome, be it binary or continuous, or markers in linkage disequilibrium (LD) with those causal loci. Increasingly, however, gene-based tests are coming to the forefront, especially as sequencing technologies mature and grow cheaper [1-3]. Gene-based tests are useful to provide insight into whether a region of the genome has a significant association with some outcome and for inter-gene significance comparisons, despite differences in the size of genes [4,5]. Development of such tests is difficult, however, as markers are usually correlated with one another and have highly variable minor allele frequencies [6]. As a result, tests have often been born more out of practicality or computational ease. Some gene-based tests take the smallest p-value over all the markers in the region. Others, such as that implemented in PLINK, take a more sophisticated approach, converting p-values of markers to $\chi_{1}^{2}$ test statistics, averaging those tests statistics, then comparing it to a null distribution generated from permutations of the outcome under the null [1,7]. Liu et al. (2010) use a similar, though more efficient method, in which they again convert marker p-values to $\chi_{1}^{2}$ test statistics, take the sum of those test statistics, then generate a null distribution by sampling from sums of correlated $\chi_{1}^{2}$ random variables. Both approaches are intuitive and valuable ways to assess gene significance, though in both cases the power for detection of a gene becomes not only a function of the effect size of the individual markers, but the degree to which markers are in LD with one another. For example, assuming only one marker in the region has a truly non-zero effect size, power for detecting that effect will be higher if the marker is in high LD with other markers than if it is independent of them. Moskvina et al. (2012) independently made this observation, having noticed that the significance of regions they tested changed according to how much they pruned markers in high LD with one another [8].

One way to think about why this phenomenon occurs is that, rather than transforming the test statistic so that markers highly correlated with one another “mean less” because they do not contribute independent information, they transform the null distribution for certain markers under the null to “mean more.” As a result, the type 1 error is maintained, but power varies as a function of the correlation between the marker and surrounding markers. Intuitively, this is not a desirable property because it will lead to a systematic under-detection of those loci that happen to be independent of proximal markers even though they are inherently no less important in predicting the outcome. This issue becomes particularly problematic for sequence data since there would generally be even more correlation. However, the issue is a zero-sum trade-off; what results in less power for detection of single nucleotide polymorphisms (SNPs) in low LD translates to more power for detection of SNPs in high LD. Though, if there is an underlying common function or characteristic of those genomic regions whose significant SNPs are not in high LD, perhaps due to when they first occurred in evolutionary history, such regions will likely be missed in association analyses so that potentially key regions will not be studied in greater depth. As a result of this shortcoming, which may be more or less important depending on the specific LD structure of the genomic region under study, we propose two methods, one of which transforms summary Z-statistics from univariate regressions of markers so that it follows a standard parametric distribution under the null hypothesis and power does not vary with the LD structure, and the second of which uses an Eigen decomposition of the information matrix to find the “effective” amount of information in the region and increases power by performing a more parsimonious test. If the information matrix is evaluated under the null, this latter test is essentially a dimension-reduced score test analogue to a method described in [2,3], which finds the principal components of the data matrix. Specifically, for the first approach we propose, we find Z-statistics associated with each marker in our region and the correlation matrix of the markers and perform a *χ*^2^ test, an approach similar to that proposed by Yang et al. (2012) [9]. In case the correlation matrix is imperfectly known, we propose a modification of this test that adjusts the correlation structure to protect the type 1 error. In the latter approach, we calculate the eigenvectors associated with the information matrix to obtain a most powerful linear combination of the scores, on which we again perform a *χ*^2^ test after having normalized by the variance of the loadings. These two approaches are proposed for different situations: the dimension reduction approach for when there is correlation between markers and the analyst has access to the original data, and the summary-statistic test for when the analyst only has access to Z-statistics from univariate regressions of the outcome on the marker along with an estimate of the correlation matrix of the markers in the population from which they come. Moskvina et al. (2012) also propose solutions, one of which is based on Hotelling’s *T*^2^ test, while another is based on multivariate logistic regression, though concludes that both perform similarly. We compare these various approaches under different structures of LD and effect size. We apply our methods to a case-control sequence data set of oral cleft and an already-published GWAS study of Chronic Obstructive Pulmonary Disorder (COPD) [10].

## Methods

### Description of permutation-based gene tests

First we show how power differs for permutation-based gene tests as a function of linkage disequilibrium from a theoretical perspective. When we refer to permutation-based gene tests, we mean gene-based tests in which the sum of the *χ*^2^ statistics for markers is taken and then an empirical p-value is calculated by permuting case-control status to generate a null distribution. By Imhof (1961), in connection with Liu et al. (2010), we know that the null distribution of the permutation-based test is $\overset{q}{\underset{i=1}{\sum }}\lambda_{i}\chi_{1}^{2}$, where $\chi_{1}^{2}$ is a chi-squared random variable on 1 d.f., *λ*_*i*_ is the *i*^*t* *h*^ eigenvalue of *Σ*, the *q*×*q* correlation matrix of the SNPs comprising the gene to be tested. Under the alternative, the distribution is approximately $\overset{q}{\underset{i=1}{\sum }}\lambda_{i}\chi_{1}^{2}(\delta_{i}^{2})$, where the non-centrality parameter *δ*_*i*_ is calculated $\delta_{i}=v_{i}^{t}\cdot \mu /\sqrt{\lambda_{i}}$, where *v*_*i*_ is the eigenvector of *Σ* corresponding to *λ*_*i*_, and ***μ*** is the q-dimensional mean of the multivariate normal distribution of Z-statistics calculated for univariate regressions of each SNP. ***μ*** is a function of the power for detection of each SNP in the gene. The distribution under the alternative is approximately $\overset{q}{\underset{i=1}{\sum }}\lambda_{i}\chi_{1}^{2}(\delta_{i}^{2})$, rather than exactly, because the correlation matrix of marker Z-statistics coming from univariate regressions diverges from the correlation structure of the covariates when under the alternative. However, so long as there is not significant variation between observations in the true probability of being a case, this divergence will not be relevant. Since the relative risk of disease conferred by most minor alleles is small, it is likely that the approximation is valid in most studies.

Suppose there is a single causal SNP *X*_1_ and, without loss of generality, it is the first entry in the (*q*+2)-marker gene and *q* other SNPs, *Z*_*i*_, 1≤*i*≤*q*, are correlated with it but do not cause the outcome. Also assume that the correlation coefficient between *X*_1_ and *Z*_*i*_ is *ρ*_*i*_, and the last SNP, *X*_2_, is uncorrelated with *X*_1_ and does not cause the outcome. The first entry of ***μ***, which represents the mean of the Z-statistic for *X*_1_, can be written $k_{1}\cdot \sqrt{n}$ for some number *k*_1_ where *n* is the sample size. Since the asymptotic relative efficiency for using *Z*_*i*_ rather than *X*_1_ is $\rho_{i}^{2}$, the *i*^*t* *h*^ entry of ***μ***, that associated with *Z*_*i*_, can be written $k_{1}\cdot \rho_{i}\cdot \sqrt{n}$[11,12]. The entries of *μ*_1_, the (*q*+2)-dimensional mean of the Z-statistics corresponding to the permutation-based gene test when the causal SNP is *X*_1_, are

$${\mu_{1}}^{T}=(k_{1}\sqrt{n},\;\;k_{1}\cdot \rho_{1}\sqrt{n},\;\;k_{1}\cdot \rho_{2}\sqrt{n},\ldots ,\;\;k_{1}\cdot \rho_{q}\sqrt{n},\;\;0).$$

In contrast, suppose that the correlation structure among SNPs is the same, that is, Cor(*X*_1_,*Z*_*i*_)=*ρ*_*i*_ for 1≤*i*≤*q*, but *X*_1_ does not cause the outcome, and instead *X*_2_, the SNP uncorrelated with all other (*q*+1) markers, causes the outcome and to the same degree as *X*_1_ did so in the previous scenario. Then *μ*_2_, the (*q*+2)-dimensional mean in this case is

$${\mu_{2}}^{T}=(0,\;\;0,\;\;0,\ldots ,\;\;0,\;\;k_{1}\sqrt{n}).$$

**Figure 1:**
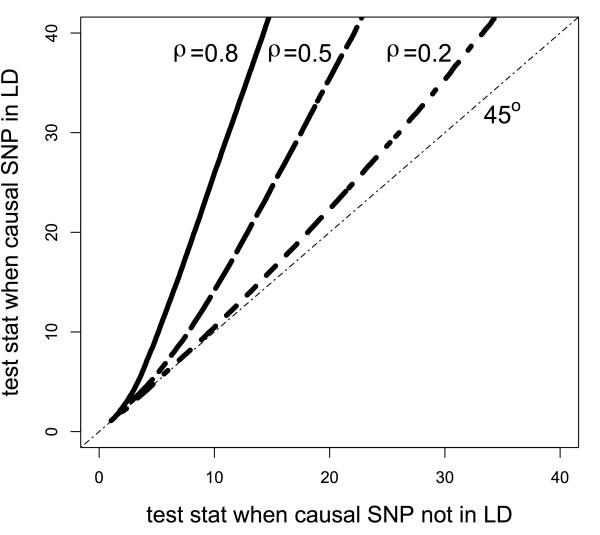
**Q-Q plot of permutation-based gene test statistic under alternative for correlated region versus uncorrelated region.** The higher the correlation in the LD block containing the causal SNP, the more power relative to the causal SNP not in the LD block using the permutation-based test. Lines are labelled with the correlation coefficient for SNPs in the block.

If $Q\equiv \overset{q}{\underset{i=1}{\sum }}\lambda_{i}\chi_{1}^{2}(\delta_{i}^{2})$, the power of an size *α* (i.e., type 1 error rate) permutation-based gene test is *P*(*Q*>*c*^∗^), where *c*^∗^ is the (1-*α*) quantile of the random variable $\overset{q}{\underset{i=1}{\sum }}\lambda_{i}\chi_{1}^{2}$. The intuition behind the power gains for causal SNPs in regions of LD is that the non-centrality parameters *δ*_*i*_ will generally be larger when the causal SNP is in a region of LD than when it is not. Providing greater rigor than this intuition is difficult because the calculation of *δ*_*i*_ for all 1≤*i*≤(*q*+2), even in the simple case of *ρ*_*i*_=*ρ*_*j*_ for all 1≤*i*≤*q*, can be complicated. However, sampling from the appropriate distributions demonstrates that there is greater power to detect a gene-outcome association when the causal SNP is in a region of LD. Figure 1 shows that under the alternative of gene-outcome association and for a fixed effect size and *ρ*_*i*_=*ρ*_*j*_=*ρ* for 1≤*i*≤*q*, i.e., the (*q*+2)×(*q*+2) correlation matrix ***Σ*** is

$$\Sigma =\left[\begin{array}{cccccc} 1 & \rho & \rho & \ldots & \rho & 0\\ \rho & 1 & \rho & \ldots & \rho & 0\\ \rho & \rho & 1 & \ldots & \rho & 0\\ \vdots & \vdots & \vdots & \ddots & \vdots & \vdots \\ \rho & \rho & \rho & \ldots & 1 & 0\\ 0 & 0 & 0 & \ldots & 0 & 1 \end{array}\right],$$

the distribution of the test statistic *Q* when the causal SNP in the gene is in a region of LD is stochastically greater than the distribution when the causal SNP in the gene is not in a region of LD. As a result, power is greater to detect a gene whose causal SNP is in a region of LD for a test of any size *α* in a permutation-based gene test. Figure 1 was generated assuming a gene consisting of 7 markers, where 6 markers were correlated with coefficient *ρ*, shown for values 0.2, 0.5, and 0.8 in the figure, with the causal SNP in the LD block (y-axis) versus not in the LD block (x-axis). While the example may seem contrived, if we consider *q*=10 so that our gene consists of 12 SNPs in total, the correlation structure in this example is similar to choosing the first 12 SNPs of BRCA1 [13], in which case *ρ*≈0.96, and where the last row and column above would be approximately 0.24 rather than 0. As a result, the variation in power observed when using permutation-based tests may have real-world import on the groupings of SNPs able to be detected. 

**Figure 2:**
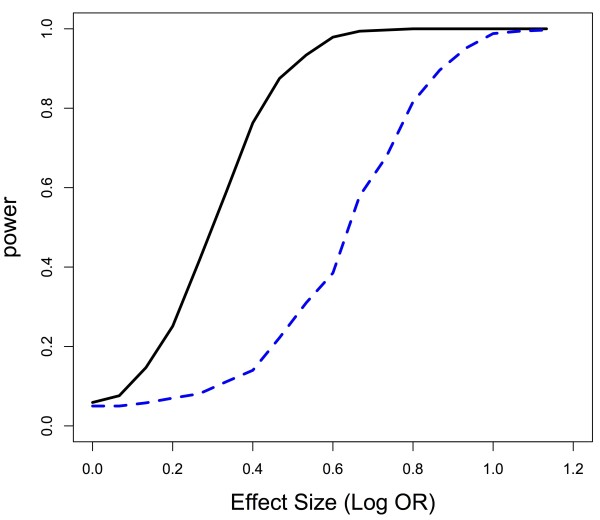
**Power for permutation-based gene test as a function of causal SNP’s location relative to correlated region and effect size.** There is more power to detect a SNP in high LD with other, non-causal SNPs (solid, black line), than a SNP in low LD (dashed, blue line) for an identical effect size.

**Figure 3:**
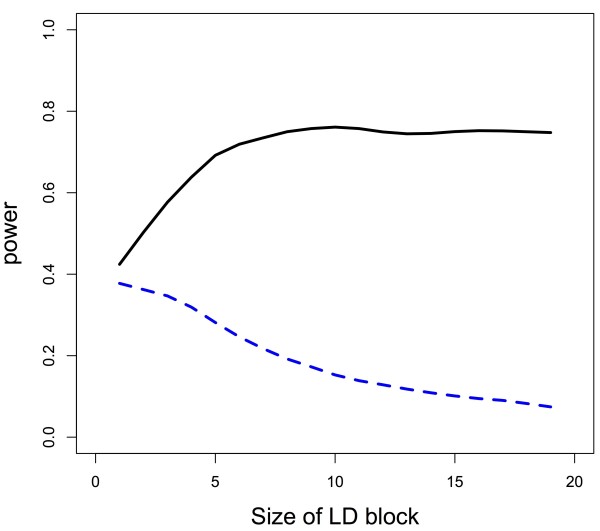
**Power for permutation-based gene test as a function of causal SNP’s location relative to correlated region and size of LD block.** For a constant effect size, size of the LD block in which the causal SNP is (or is not) located is related to the power for detection. The black, solid power curve refers to the permutation-based gene test when the causal SNP is in the LD block, and the blue, dashed power curve refers to the test when the causal SNP is not in the LD block.

Figures 2 and 3 demonstrate from a graphical perspective how permutation-based gene tests can have variable power as the LD structure changes. The figures illustrate that there is more power for the permutation-based gene test when causal SNPs are in high LD blocks as compared to causal SNPs in low LD blocks. Additionally, if a causal SNP is not in LD with other SNPs, but large LD blocks exist in the gene, power for the permutation-based gene test decreases as the size of the block increases. Data were generated with a minor allele frequency of all SNPs of approximately 0.3 with Hardy-Weinberg equilibrium assumed, and, within the LD block, correlation between SNPs was approximately 0.65, whereas SNPs not in the LD block were independent of one another. The gene consisted of 20 SNPs, and there were 600 subjects with an equal number of cases and controls. Power calculations were based on 1000 iterations at each effect size (Figure 2) or LD block size (Figure 3). We calculated power at 18 different effect sizes (Figure 2), with the effect size ranging from a log odds ratio (OR) of 0 to 1.2, and 20 different LD block sizes (Figure 3), with the LD block size ranging from 1 SNP to 20 SNPs. So when the size was 20 SNPs, the LD block was the entirety of our hypothetical gene (Figure 3). Binary outcomes were generated assuming a logistic regression model, where the presence of the causal SNP determined probability of being a case or control. Simulations assumed a single causal marker to clearly illustrate how power changes as a function of location of the SNP within the LD structure, though as the theoretical work above shows, the result holds for any number of causal markers.

As described in the Additional file 1 section of the manuscript, the authors have posted an R script online that allows one to see power variation for permutation-based gene tests as a function of the correlation structure, power to detect the causal marker (in a univariate regression), and its location relative to the other SNPs comprising the gene.

### Description of summary statistic based test

We first describe a simple solution to the problem of how LD structure can affect the power to detect genomic regions in which there are significant SNPs. Our solution is based on the Z-statistics associated with each marker and the correlation matrix of the SNPs. Since we propose this test as one that can be used without a full data set, we propose a modification of it in case the true correlation structure is not perfectly known or it is believed that study participants are not reflective of the population from which the correlation of SNPs are calculated (such as with HapMap reference panels).

One solution to the problem of under-detection of SNPs in low LD posed by permutation tests is transformation of the gene-based test statistic so that under the null it follows a standard parametric distribution, rather than creating a non-standard null distribution through permutations. One way to accomplish this task, and one in which it is unnecessary to reanalyze data, is to perform a joint test on the Z-statistics coming from a univariate regression model for each marker. It is a an approach similar to that described by Yang et al. (2012), though uses summary statistics directly rather than estimated model coefficients. Since the estimated covariance structure of these statistics under the null is the correlation of the markers themselves [14], one can use the data to estimate the covariation of the Z-statistics or an online database of LD or correlation structure of SNPs. The intuition behind this result is that if two markers are highly correlated, then when by chance under the null, one marker is significant (or insignificant), the other marker will similarly be significant (or insignificant). However, if two markers are not correlated, then the chance significance or insignificance of one marker will not inform the significance of the other marker. And since Z-statistics have variance 1 by definition, their covariance matrix is identical to their correlation matrix. Thus, supposing we have *q* markers, which, from previous studies, are known to have Z-statistics of ***Z*** =(*Z*_1_,…,*Z*_*q*_)^*T*^, and which have correlation structure ***V***, then under the null hypothesis of no marker being associated with the outcome, $T\equiv {\text{Z}}^{T}V^{-1}\text{Z}∼X_{q}^{2}$. One then rejects the null of no association between the region composing the *q* markers and the outcome for an extreme value of the test statistic *T* using a pre-determined *α* level.

If one is not confident that *V* accurately reflects the correlation of the SNPs in the data matrix and therefore ***Z*** under the null hypothesis (because *V* is an estimate possibly coming from a reference panel if the original data is not available and the analysis is performed only with access to summary Z-statistics), it is possible to construct a more conservative test by shrinking the off-diagonal elements of *V* towards 0. Thus, if *V* is an estimate of the covariance of the SNPs, one can compute $V_{\gamma }^{*}\equiv \mathrm{\gamma V}+(1-\gamma ){\text{I}}_{q}$, where ***I***_*q*_ is a *q*×*q* identity matrix and 0≤*γ*≤1.

Again using [15], if *Z*∼*MVN*(0,*V*) but we use $V_{\gamma }^{*}$ as an estimate of the correlation structure in the gene based test, then $Z^{T}V_{\gamma }^{*-1}Z∼\overset{q}{\underset{i=1}{\sum }}\lambda_{i}\cdot \chi_{1}^{2}$ where *q* is the dimension of vector *Z*, and where *λ*_*i*_ is the *i*^*t* *h*^ eigenvalue of $VV_{\gamma }^{*-1}$. By construction of $V_{\gamma }^{*-1}$, $\overset{q}{\underset{i=1}{\sum }}\lambda_{i}<\dim(Z)$ for 0<*γ*<1, where dim(·) the dimension of the vector argument. This fact in itself does not not necessarily imply a more conservative test for all size *α* tests because when eigenvalues are not equal to one another as is the case for the decomposition of $VV_{\gamma }^{*-1}$ with $V\neq V_{\gamma }^{*-1}$, $\overset{k}{\underset{i=1}{\sum }}\lambda_{i}<\dim(Z)$ can be true, but $Z^{T}V_{\gamma }^{*-1}Z$ is not stochastically less than $\chi_{\dim(Z)}^{2}$, the null distribution of the test statistic when the correlation structure is correctly known. However, for modest values of *γ* (i.e., 0.8-1.0, where 1.0 corresponds to no transformation), the test using the adjusted correlation matrix will generally be more conservative. It is difficult to obtain simple solutions for how much conservative a test will be using this modification since it will depend on the quantile corresponding to the intended type 1 error and the specific $VV_{\gamma }^{*-1}$.

Thus, to give a practical sense of useful values of *γ*, we borrowed a correlation structure of eight SNPs in the INS-IGF2 gene of Chromosome 11 from the CEU reference panel in one case and the CHB+JPT reference panel in the other case [13]. If the true, underlying population giving rise to the SNPs was more reflective of the CEU reference panel, but the analyst incorrectly guessed the correlation structure to be that of the CHB+JPT reference panel when performing the summary statistic gene-based test,the type 1 error rate for a nominal 0.05 size test would in fact be a highly inflated 0.61. Similarly, if the true, underlying population giving rise to the SNPs was more reflective of the CHB+JPT reference panel, but the analyst incorrectly guessed the correlation structure to be that of the CEU reference panel for the summary statistic gene-based test, the type 1 error rate for a nominal 0.05 size test would be 0.69. If the type 1 error is inflated in one scenario, there is no implication that it will be deflated in the ’inverse’ scenario.

In the scenario where the underlying population was more reflective of the CEU panel, the type 1 error using our modified summary statistic test with adjusted correlation matrix and *γ*’s of 0.9, 0.5, and 0.3 led to reduced error rates of 0.36, 0.11, and 0.09, respectively, instead of 0.61. When the underlying population was CHB+JPT, the type 1 error using our adjustment correlation matrix and *γ*’s of 0.9, 0.5, and 0.3 led to reduced error rates of 0.45, 0.10, and 0.07, respectively, instead of 0.69.

Type 1 error rates were calculated by sampling from an eight-dimensional multivariate normal distribution with mean vector ***0*** (i.e., under the null) and unit variance for all elements, but whose correlation structure corresponded to the “true” correlation structure of the population in any one of these two situations described, be it that of the CEU or CHB+JPT panel. We then multiplied each sample, ***Z***_***1***_,… ***Z***_***n***_, with *n*=2000, by the estimated correlation matrix, *V*_*γ*_, by taking $Z_{i}^{T}V_{\gamma }^{-1}Z_{i}$, where *V* was taken from the panel unreflective of the truth (e.g., the CEU correlation structure was used for an underlying, true correlation structure corresponding to the CHB+JPT panel and vice versa) and possibly modified with the *γ* parameter. To find the error rate, we calculated the probability mass beyond the 0.95 quantile of $\chi_{8}^{2}$, the distribution $Z_{i}^{T}V_{\gamma }^{-1}Z_{i}$ would follow if *Var*(*Z*_*i*_)=*V*_*γ*_ (i.e., assuming the true correlation of ***Z***_***i***_ was known).

While in all cases, the nominal size of the test is not quite achieved, type 1 error is greatly reduced, and in some cases will be achieved when divergence between correlation structures of the true and hypothesized populations are not as great as that in these scenarios. The greatest reduction in type 1 error occurs with initial deviation of *γ* from 1; i.e., a movement of *γ* from 1 (indicating an unadjusted correlation matrix) to 0.9 will reduce type 1 error more than a movement of 0.6 to 0.5. And, as mentioned, with very small values of *γ*, there is not necessarily a guarantee of continued reduction in type 1 error for some nominal *α* level tests, nor should such *γ* values be used if indicative of no confidence in one’s estimated correlation matrix.

To simulate a less drastic divergence between true and estimated correlation matrices and assess error rates and the proposed adjustment method in that context, we generated correlation matrices whose entries were beta-distributed random variables with means corresponding to the entries in the CHB+JPT reference panel and standard deviation approximately 0.03-0.04 (approximately because standard deviation is partly a function of the mean). With a population whose underlying correlation structure was in truth reflective of the CHB+JPT panel, but using the generated correlation matrices in our calculations of the test statistic, the average type 1 error rate was 0.19. Adjusting the generated correlation matrices according to our method and with a *γ* value of 0.95, the error rate was reduced to 0.05. Adjustment of the generated correlation matrices with a *γ* value of 0.90 led to a type 1 error rate of 0.03.

The summary statistic based test we have proposed is a viable way of performing gene-based testing when one does not want power to vary as a function of the correlation structure of the SNPs composing the gene. A weakness of such an approach is an inability to know the underlying correlation structure of the SNPs used in the univariate regression analyses giving rise to the Z-statistics used in the summary statistic test. We have shown that incorrect guesses of the underlying correlation structure can lead to a significant increase in the type 1 error rate and therefore have proposed an adjustment method which can lead to achievement of error rates in line with the nominal size of the test. However, since by supposition of this setting the correlation structure of SNPs is never known, it is impossible to know the needed value of *γ*. As a result, it may be best to perform one’s summary statistic based test with *γ* values ranging from 0.8-1.0 as a sensitivity analysis to see how one’s conclusions change based on different values. Values of *γ* smaller than 0.8 probably reflect little confidence in the estimated correlation structure, in which case feasibility of the analysis in the first place should be reassessed.

### Description of Eigen decomposition-based test

The above approach controls for the LD structure of the region under study by transforming the test statistic so that LD no longer affects the power to detect significant regions. However, there are other ways one can make use of the LD structure to construct more powerful tests, such as by dimension reduction. Consider an extreme example where an investigator is interested in a region with *d* SNPs, and these SNPs are in nearly perfect LD so that a correlation matrix of them has off-diagonal entries close to 1. Because they are highly correlated, the association between any SNP and the outcome adds little information on top of that between any other SNP and the outcome. As a result, intuition may tell us that using a *d*-d.f. test on the region after having properly accounted for the underlying LD structure is not the most powerful approach since there is essentially the information of 1 SNP contained in the entire region. On the other hand, it is difficult to justify focusing on any one SNP over another as one might do when “tagging” the region. Also, while no additional SNP contributes much information over another, there is still some amount of additional information contained in each one that, ideally, would not be ignored.

Finding the eigenvectors and values of the information matrix is one way to approach this scenario. Unlike the previously proposed summary statistic test, this analysis approach requires the original SNP data. It gleans the essential information from the LD block, thus stripping away extraneous information that dilutes the power of proposed tests while avoiding the arbitrariness of pruning the number of SNPs being examined. It is an approach similar to finding the principal components of the data matrix and then regressing the outcome on those components if the information matrix is evaluated under the null [2,3] and may even be thought of as a score test analogue to it. If certain covariates have been shown to control for population stratification, it also may be fitting for the matrix to be evaluated under the alternative using the estimated effect sizes of those covariates. Also, as simulations demonstrate, there may be power gains under certain correlation structures or when effect or sample sizes are small. Since the information matrix is the covariation of the scores associated with each marker and since score functions of highly correlated markers are correlated as well, identifying the chief axes of the covarying scores is synonymous with finding the eigenvectors of the information matrix. One can then detect small deviations from the mean under the null hypothesis, the ***0*** vector, by performing a parsimonious test. Additionally, if we are considering the underlying model to be that of logistic regression, both the information matrix and the score have simple forms and are computationally tractable.

We now describe how to construct the test, which will place no constraint on estimation of the intercept, but reduce the dimension of the covariates underlying the scores. The score function associated with the *j*^*t* *h*^ marker under a logistic regression model is

$$\;S(\beta_{j})=\frac{\mathrm{\partial L}(\beta )}{\partial \beta_{j}}=\underset{i}{\sum }y_{i}x_{\text{ij}}-\underset{i}{\sum }n_{i}x_{\text{ij}}\frac{\exp(\underset{k}{\sum }\beta_{k}x_{\text{ik}})}{1+\exp(\underset{k}{\sum }\beta_{k}x_{\text{ik}})}.$$

While the (*j*,*l*)^*t* *h*^ entry in the information matrix is

$$\begin{array}{lll} I_{q+1\;(j,l)} & =\text{Cov}(S(\beta_{j}),S(\beta_{l}))=\text{Cov}(\underset{i}{\sum }y_{i}x_{\text{ij}},\underset{i}{\sum }y_{i}x_{\text{il}})\; & \;\\ & =\underset{i}{\sum }x_{\text{ij}}x_{\text{il}}P(y_{i}=1)\{1-P(y_{i}=1)\}\; & \;\\ & =\underset{i}{\sum }x_{\text{ij}}x_{\text{il}}\frac{\exp(\overset{k}{\underset{m=0}{\sum }}\beta_{m}x_{\text{im}})}{{\left\{1+\exp(\overset{k}{\underset{m=0}{\sum }}\beta_{m}x_{\text{im}})\right\}}^{2}},\; & \; \end{array}$$

where we could estimate *P*(*y*_*i*_=1) under an intercept-only model, i.e., the proportion of cases, or another model that included potential confounders. For the sake of explanation, we will proceed as if using the intercept-only model. The information matrix for the logistic regression model is then

$$I_{q+1}=\left[\begin{array}{cccc} I_{q+1\;(1,1)} & I_{q+1\;(1,2)} & \ldots & I_{q+1\;(1,q+1)}\\ I_{q+1\;(2,1)} & I_{q+1\;(2,2)} & \ldots & I_{q+1\;(1,q+1)}\\ \vdots & \vdots & \ddots & \vdots \\ I_{q+1\;(q+1,1)} & I_{q+1\;(q+1,2)} & \ldots & I_{q+1\;(q+1,q+1)} \end{array}\right],$$

where, as is consistent from our definition of *I*_*j*,*l*_, *I*_*l*,*j*_=*I*_*j*,*l*_. Also, *I* is (*q*+1)×(*q*+1) because there are *q* markers and 1 intercept available for use in the model.

Now define ***I***_***q***_ to be the *q*×*q* information matrix for the covariates, not including the intercept. That is, ***I***_***q***_ is ***I***_***q+1***_ without the first column and first row of ***I***_***q+1***_. ***I***_***q***_ can be decomposed into ***E*** ***W*** ***E***^***-1***^, where ***E*** ≡(***e***_***1***_, ***e***_***2***_, ⋯, ***e***_***q***_) is a matrix of the *q* eigenvectors ***e***_***i***_, 1≤*i*≤*q*, and ***W*** ≡diag(*λ*_1_, *λ*_2_ ⋯,*λ*_*q*_) is a diagonal matrix of the corresponding eigenvalues *λ*_*i*_, 1≤*i*≤*q*. While one can use a variable number of eigenvectors in the analysis, if we suppose that we are in the situation described above where all *d* markers are highly correlated, then making use of just the first component may be sufficient to adequately encompass the information contained in the genomic region. More generally, a systematic criterion for deciding which eigenvectors to use is employing all those whose associated eigenvalues are larger than the average eigenvalue.

For the sake of explanation, we suppose first that we will construct the test using only the eigenvector associated withe largest eigenvalue and then generalize later. Denote ***e***_***1***_ the first column of ***E*** and vector associated with the largest eigenvalue (assume the columns of ***E*** are ordered according to decreasing eigenvalue). The interpretation of ***e***_***1***_ is the axis of maximum variation of the distribution whose covariance matrix is ***I***_***q***_, and *λ*_1_, the associated eigenvalue, can be interpreted as the variation along that axis. Since ***I***_***q***_ is *q*×*q*, ***e***_***1***_ is a (*q*×1) unit eigenvector. Define a new 2×2 information matrix ***I***^***∗***^ as ***I***^***∗***^_(1,1)_≡**I**_*q*+1 (1,1)_, ***I***^***∗***^_(2,2)_≡*λ*_1_, and ***I***^***∗***^_2,1_= ***I***^***∗***^_1,2_= ***e***_***1***_^*T*^· ***I***_***q (,1)***_, where ***I***_***q (,1)***_ is the first column of ***I***_***q***_, ***v***^*T*^ denotes the transpose of vector ***v***, and ***e***_***1***_^*T*^· ***I***_***q (,1)***_ denotes the dot product of vectors ***e***_***1***_ and ***I***_***q (,1)***_. The test statistic for the 1 d.f. score test analogue of the method described in [2,3] is then (**S**^*t*^· ***e***_***1***_)^2^·[(***I***^∗ -1^)_(2,2)_], where ***S*** is the *q*-dimensional vector of scores associated with the *q* markers, which follows a $\chi_{1}^{2}$ distribution under the null hypothesis of no gene-outcome association.

To generalize the method to using *p* eigenvectors, similar to regressing the outcome on the first *p* principal components of the data matrix, again perform an Eigen decomposition of ***I***_***q***_, and define ***e***_***1***_,…, ***e***_***p***_ as the *p* unit eigenvectors of length *q* associated with the *p* largest eigenvalues. Call those associated eigenvalues *λ*_1_,…,*λ*_*p*_. Define a new information matrix ***I***^***∗∗***^ as ***I***^***∗∗***^_(1,1)_= ***I***_***q+1 (1,1)***_, ***I***^***∗∗***^_(*m*,*m*)_=*λ*_*m*_ (where 2≤*m*≤*p*), ***I***^***∗∗***^_(*m*,*n*)_= ***I***^***∗∗***^_(*n*,*m*)_=0 (where *m*≠*n* and 2≤*m*,*n*≤*p*), and ***I***^***∗∗***^_(*m*,1)_= ***I***^***∗∗***^_(1,*m*)_= ***e***_***m***_^*T*^· ***I***_***q (,1)***_ (where 1<*m*≤*p* and ***I***_***q (,1)***_ denotes the first column of ***I***_***q***_*Y*). Note that the off-diagonals of ***I***^***∗∗***^ which are neither the first row nor first column are zero by the orthogonality of eigenvectors; i.e., for 1<*m*,*n*≤*p*, *m*≠*n*, ${I^{**}}_{\left(m,n\right)}={I^{**}}_{\left(n,m\right)}=e_{n}^{T}\cdot I_{q}\cdot e_{m}=e_{m}^{T}\cdot I_{q}\cdot e_{n}=0$. ***I***^***∗∗***^ is (*p*+1)×(*p*+1) and looks as follows

$$\begin{array}{l} \;I^{**}=\left[\begin{array}{ccccc} I_{q+1\;(1,1)} & {e_{1}}^{T}\cdot I_{q\;(,1)} & {e_{2}}^{T}\cdot I_{q\;(,1)} & \ldots & {e_{p}}^{T}\cdot I_{q\;(,1)}\\ {e_{1}}^{T}\cdot I_{q\;(,1)} & \lambda_{1} & 0 & \ldots & 0\\ {e_{2}}^{T}\cdot I_{q\;(,1)} & 0 & \lambda_{2} & \ldots & 0\\ \vdots & \vdots & \vdots & \ddots & \vdots \\ {e_{p}}^{T}\cdot I_{q\;(,1)} & 0 & 0 & \ldots & \lambda_{p} \end{array}\right] \end{array}$$

Define ${I^{**}}_{p\times p}^{-1}$ as the lower-right *p*×*p* sub-matrix of ***I***^***∗∗***^^-1^. The test statistic is $S^{T}\cdot (e_{1}\;\ldots \;e_{p})\cdot {I^{**}}_{p\times p}^{-1}\cdot {\left(e_{1}\;\ldots \;e_{p}\right)}^{T}\cdot S$, which follows a $\chi_{p}^{2}$ distribution under the null hypothesis of no gene-outcome association, where again ***S*** is a vector of scores of length *q*.

Oftentimes in GWAS, population stratification can obscure the relationship between markers (or groups of markers) and outcomes. In these settings, it is necessary to account for stratification by fitting models with covariates or ancestry informative markers (AIM) that adjust for the different populations composing the sample. Reducing the dimension of such covariates along with the markers making up the gene renders them less effective if not useless for their intended purpose of controlling for population stratification. Thus, it is necessary to construct a score test where only a chosen subset of the covariates have their dimension reduced and the information matrix is evaluated under the alternative for those covariates whose dimension is not reduced. Doing so is not difficult and only requires treatment of the adjusting covariate in the quasi-information matrix as we treated the intercept in ***I***^∗∗^, where the off-diagonal entries were a linear combination of the appropriate eigenvector and *q*-length sub-column of the original information matrix. So suppose there are *q* markers and we only want to reduce the dimension of the last (*q*-1) of this group. Let ***I***_***(q+1)***_ be the (*q*+1)×(*q*+1) information matrix and define ***I***_***(q-1)***_ as the lower right (*q*-1)×(*q*-1) sub-matrix of ***I***_***(q+1)***_. Decompose ***I***_***(q-1)***_ into ***E***^***′***^ ***W***^***′***^ ***E***^***′***^^***-1***^, where ***E***^***′***^ is the matrix of (*q*-1) eigenvectors, *ej*′ for 1≤*j*≤(*q*-1), of ***I***_***(q-1)***_ and ***W***^***′***^ is the diagonal matrix of corresponding eigenvalues, *λj*′ for 1≤*j*≤(*q*-1), and we use ***E***^***′***^ and ***W***^***′***^ to differentiate these matrices from those defined above and *not* to indicate the transpose of these matrices. Suppose we want to use the first *p*^′^ eigenvectors for our test of the (*q*-1) markers in the group whose dimension we reduce and where *p*^′^≤(*q*-1). The quasi-information matrix is defined

$$\begin{array}{lll} & \;I^{***}=\left[\;\begin{array}{ccccc} I_{q+1\;(1,1)} & I_{q+1\;(1,2)} & {e_{1}^{\prime }}^{T}\cdot I_{q-1\;(,1)} & \ldots & {e_{p^{\prime }}^{\prime }}^{T}\cdot I_{q-1\;(,1)}\\ I_{q+1\;(2,1)} & I_{q+1\;(2,2)} & {e_{1}^{\prime }}^{T}\cdot I_{q-1\;(,2)} & \ldots & {e_{p^{\prime }}^{\prime }}^{T}\cdot I_{q-1\;(,2)}\\ {e_{1}^{\prime }}^{T}\cdot I_{q-1\;(,1)} & {e_{1}^{\prime }}^{T}\cdot I_{q-1\;(,2)} & \lambda_{1}^{\prime } & \ldots & 0\\ \vdots & \vdots & \vdots & \ddots & \vdots \\ {e_{p^{\prime }}^{\prime }}^{T}\cdot I_{q-1\;(,1)} & {e_{p^{\prime }}^{\prime }}^{T}\cdot I_{q-1\;(,2)} & 0 & \ldots & \lambda_{p^{\prime }}^{\prime } \end{array}\;\right].\; & \; \end{array}$$

Analogous to the test statistic defined in the previous test, define ${I^{***}}_{p^{\prime }\times p^{\prime }}^{-1}$ as the lower-right (*p*^′^×*p*^′^) sub-matrix of ***I***^***∗∗∗***^^-1^ and ***S***^***′***^ as the vector of scores associated with the (*q*-1) markers whose dimension we reduce. The test statistic is ${S^{\prime }}^{T}\cdot (e_{1}^{\prime }\;\ldots \;e_{p^{\prime }}^{\prime })\cdot {I^{***}}_{p^{\prime }\times p^{\prime }}^{-1}\cdot {\left(e_{1}^{\prime }\;\ldots \;e_{p^{\prime }}^{\prime }\right)}^{T}\cdot S^{\prime }$, which follows a $\chi_{p^{\prime }}^{2}$ distribution under the null hypothesis of no gene-outcome association.

While there was extended discussion of the type 1 error rate for the previous, summary statistic, test, and how it varies as a function of the shrinkage parameter, *γ*, since *V* was considered possibly misspecified, there is no such necessary discussion of type 1 error for the Eigen decomposition test; since we assume perfectly known data for the Eigen decomposition test, it is a relatively standard, parametric, hypothesis test so that asymptotic results hold and type 1 error rates correspond to the nominal size of the test.

## Results and discussion

### Simulation results for summary statistic based test and comparison with Hotelling’s ***T***^***2***^

**Figure 4:**
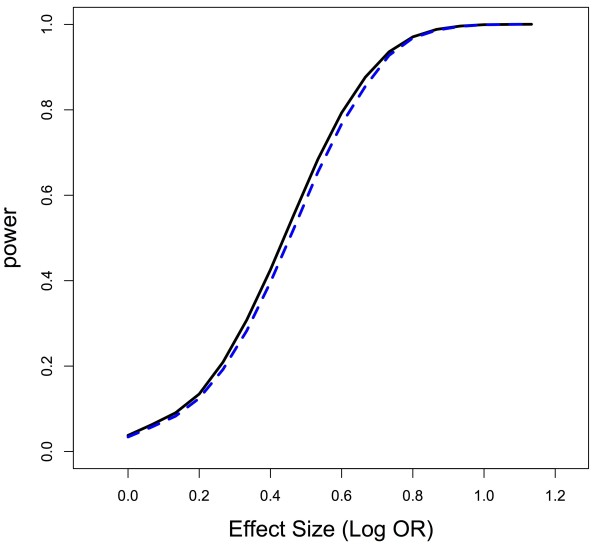
**Power comparison for a Hotelling’s T**^**2**^** approach versus summary statistic test as a function of effect size.** Power comparison of Moskvina et al.’s method (solid, black line) with our summary statistic based method (dashed, blue line) when the causal SNP is in a block of high LD. This graph looks identical to that when the causal SNP is not in a block of high LD because the power for both methods is not a function of the correlation of the SNPs and where causal SNPs are located relative to regions of correlation. Q-Q plot of permutation-based gene test statistic under alternative for correlated region versus uncorrelated region.

**Figure 5:**
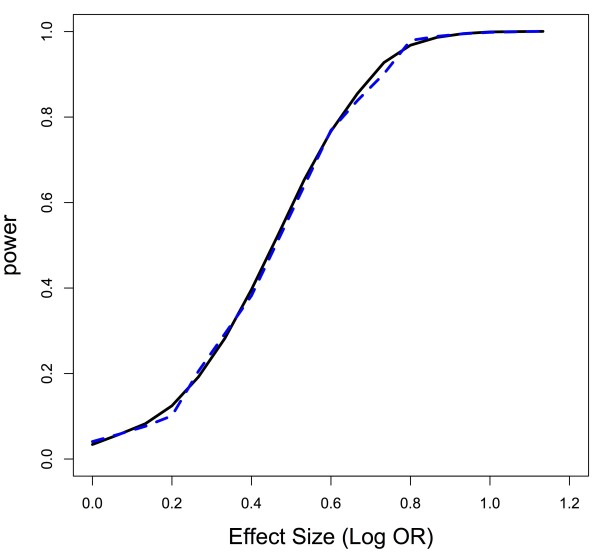
**Power comparison for summary statistic test when causal SNP is in an LD block versus not in an LD block.** A SNP in high LD with other, non-causal SNPs (solid, black line), has no more power to be detected with the summary statistic test than a SNP in low LD (dashed, blue line).

**Figure 6:**
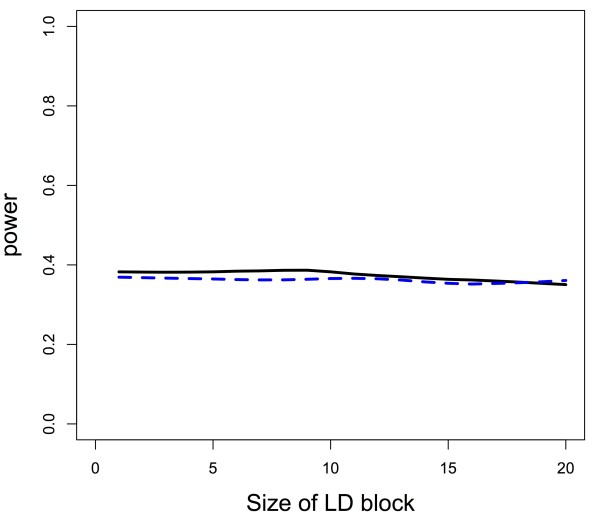
**Power comparison for summary statistic test when causal SNP is in an LD block versus not in an LD block as a function of size of the LD block.** Test working as desired since power is constant across LD block sizes for fixed effect size regardless of whether the causal SNP is in the LD block (solid, black line) or no in the LD block (dashed, blue line).

Moskvina et al. propose a test based on Hotelling’s *T*^2^. If one knows the true information matrix, it is a multivariate score test and follows a *X*^2^ distribution under the null. Supposing that there are *q* markers and ***S*** =(*S*(*β*_1_),…,*S*(*β*_*q*_))^*T*^, the associated scores, and the true information matrix is ***I***, then under the null of no marker being associated with the outcome, $S^{T}I^{-1}S∼X_{q}^{2}$. Similarly, and as described above, the summary statistic based test uses the Z-statistics associated with univariate logistic regression models, ***Z***, and the marker correlation matrix, ***V***, so that under the null hypothesis and assuming *V* is perfectly known, $T\equiv Z^{T}V^{-1}Z∼X_{q}^{2}$. While both of these approaches use similar information (i.e., some measure of SNP significance not controlling for other SNPs and an estimate relating to the correlation of those measures), in simulation the summary statistic based approach seems to have slightly less power than the Hotelling’s *T*^2^ test, but the difference is almost non-existent in many cases (Figure 4), and the summary statistic based test also seems to be more conservative, again assuming a perfectly known correlation structure *V*. Importantly, however, the summary statistic approach does not require the original, individual-level data, which is not the case with Hotelling’s *T*^2^. Power for the summary statistic based approach does not vary as a function of whether the causal SNP in the gene is in a region of LD or not (Figure 5), and, for a constant effect size, power does not vary as a function of the size of the LD block in the gene (Figure 6).

Simulations were generated under the same framework as we used with the permutation test simulation above. Covariates were generated with a minor allele frequency of approximately 0.3, and, within any LD block, correlation between SNPs was again approximately 0.65, whereas SNPs not in the LD block were independent of one another. We assumed Hardy-Weinberg equilibrium. The gene consisted of 20 SNPs, and there were 600 subjects with an equal number of cases and controls. Power calculations were based on 1000 iterations at each effect size (e.g., Figure 5) or LD block size (e.g., Figure 6). Lastly, binary outcomes were generated assuming a logistic regression model, where presence of the causal SNP determined the probability of being a case or control.

### Simulation results for Eigen decomposition-based test

**Figure 7:**
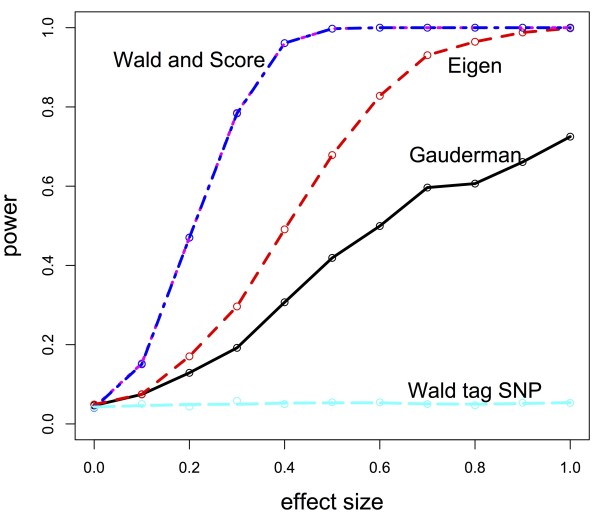
**Power comparison for different dimension-reduction tests when there is no correlation in gene.** Power comparison between the Eigen-based test (red, dashed line) and Gauderman’s method (solid, black line), along with a direct test of the causal SNPs (Wald and Score test lines are purple and blue, dashed, on top of one another) and tagging SNP under no LD (light blue, dashed line).

**Figure 8:**
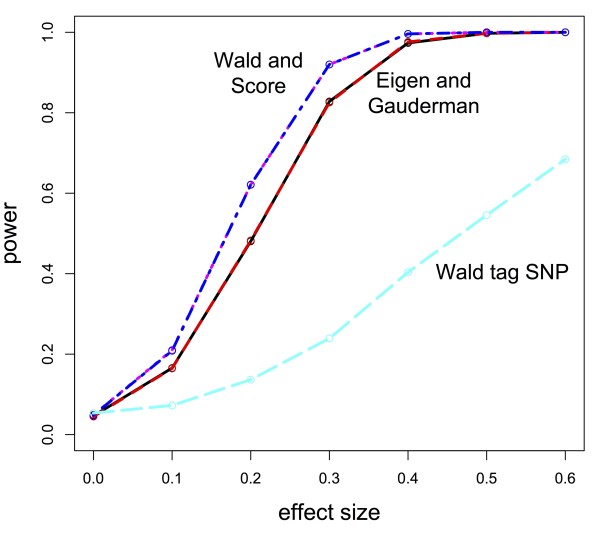
**Power comparison for different dimension-reduction tests when there is moderate correlation (*ρ*≈0*.*25) in gene.** Power comparison between the Eigen-based test (red, dashed line) and Gauderman’s method (solid, black line underneath line referring to the Eigen test), along with a direct test of the causal SNPs (Wald and Score test lines are purple and blue, dashed, on top of one another) and tagging SNP under high LD (light blue, dashed line).

We examine in simulation performance of the dimension-reduced score test when a single causal SNP was in an LD block and compare this proposed test with the method described in [2,3], in addition to 1 d.f. score and Wald tests of the causal SNP and a 1 d.f. Wald test of a tagging SNP. Figure 7 shows relative performance of these methods when there was no LD between SNPs in the gene, while Figure 8 compares methods when correlation was approximately 0.15 between any pair of SNPs in the gene. We see that the performance of the Eigen decomposition-based test performed better relative to the method proposed in [2,3] when the LD block was more weakly correlated. As the correlation increases, power of these two methods converges. Direct testing of the causal SNP, be it through a Wald or score test, performed best as expected, though of course knowledge of the true causal SNP is generally never known. Thus, we note that the Eigen decomposition-based test performs better than testing of a tagging SNP and makes unnecessary the need to decide which tagging SNP to use. In Figure 8, even under weak LD, the Eigen decomposition-based test pays little price in terms of power for no knowledge of the true causal SNP.

The simulation framework for the Eigen decomposition test differed slightly from that of the permutation-based test and the summary statistic test. The gene consisted of 15 SNPs, and there were 800 subjects with an equal number of cases and controls. Power calculations were based on 2000 iterations at each of effect size (Figures 7 and 8). We calculated power at 11 different effect sizes, with the effect size ranging from a log OR of 0 to 1.0. As described in the previous paragraph, correlation scenarios varied between independence of SNPs (Figure 7) to mutual correlation of SNPs (Figure 8). Minor allele frequency again fell in the range of “common” variants at 0.3 with Hardy-Weinberg equilibrium assumed. Binary outcomes were generated assuming a logistic regression model, and presence of the causal SNP and the effect size determined probability of being a case or control.

### Data analysis of oral cleft sequence data and COPD summary statistics

We analyze a sequence data set composed of 192 cases of cleft lip and 192 controls, on whom we have data for 14 SNPs. The data come from a GWAS in which a candidate gene was identified and then sequenced [16]. We prune the data set so that any observations with missing values or deletions are excluded, giving 172 cases and 176 controls. We also prune SNPs so that any SNP with a MAF less than 0.02 among either cases or controls is excluded, leaving 8 SNPs. We calculate the correlation matrix of SNPs by pooling cases and controls. Using the summary statistic based test, we find that the region composed of the 8 SNPs is associated with cleft lip (p=0.06). Using the Eigen decomposition based test with the two eigenvectors whose associated eigenvalue is bigger than the average eigenvalue, we calculate a p-value of 0.017; using 3 eigenvectors such that more than 80% of the variation in scores is explained, we calculate a p-value of 0.016. Thus, as is consistent with the potential power gains posed by dimension reduction, this latter test shows a stronger association between the region of 8 SNPs and cleft lip. For comparison, we also calculated a permutation test p-value, giving 0.008 (5000 permutations), and a Hotelling’s *T*^2^ p-value, giving 0.056 (non-parametric, permutation-based p-value for this test gives 0.057). Assuming little correlation among SNPs, one would expect the permutation test p-value to give a p-value similar to that of the summary statistic based test. The greater significance of the permutation test p-value suggests that a significant SNP is in LD with other SNPs and examination of the data matrix confirms this idea; a SNP whose p-value is 0.018 using a univariate logistic regression model is highly correlated with one SNP (r=0.71) and moderately correlated with another SNP (r=0.43). Since only 8 SNPs are being analyzed, these two SNPs in LD with the significant SNP may be driving the significance of the permutation test.

We also apply the summary-statistic based test to results borrowed from an already-published GWAS along with information on the correlation of markers taken from HapMap [10,13]. Pillai et al. (2009) identified 5 SNPs in the CDKAL1 gene on Chromosome 6 to be associated with Chronic Obstructive Pulmonary Disorder (COPD). We run our summary statistic based test on their results. Since the results come from a study of Norwegians, we use the (CEU) reference panel from HapMap as an estimate of the correlation structure of SNPs. The underlying population is unlikely to be identical, however, and so we adjust the correlation matrix, shrinking the off-diagonal elements toward 0 as described in the modification of the summary statistic based test to preserve the type 1 error rate. We do so with *γ* values of 1 (i.e., assuming the correlation structure is correct), 0.9, and 0.8, and corresponding p-values for the 5 d.f. test are 0.0066, 0.003, and 0.001. While the summary statistics we use are borrowed from the 100 most significant SNPs of their analysis [10], the high level of significance for tests corresponding to all *γ* values and non-arbitrary choice all SNPs in the chosen gene suggest that there is likely some association between the CDKAL1 gene and COPD. Since the test statistics are themselves random variables, specific realizations of them are not necessarily associated with increasing p-values as one would anticipate with decreases in *γ*. However, work above has shown that, in general, modest decreases in *γ* should help preserve type 1 error.

## Conclusion

With the availability of sequence data and GWAS, the importance of statistical analysis is shifting from single-locus tests to multi-loci tests that can cover genomic regions, e.g., genes or even pathways. The motivation for this development is to test a hypothesis more grounded in biology and, at the same time, to reduce the multiple testing problem and allow for many SNPs with a small effect size to increase the power of the test by their combined inclusion in the model. One of the theoretical issues that has so far not been addressed adequately is the impact of LD on the power of the test statistic in permutation-based gene tests. Controlling for the LD between loci is important to assess the relative importance of the different regions that are tested, especially when LD heterogeneity between regions is significant. In this paper, we have proposed 2 approaches that address this issue.

While our summary statistic based test may give one similar results to a Hotelling’s *T*^2^ based test, the summary statistic test does not require the original marker data from which Z-statistics are calculated. This unique advantage opens up the possibility for more in-depth analysis of previously published studies, and, with sufficient methodological development, could even suggest summary statistic based pathway analyses when combined with summary statistics from expression analyses. It also opens up the possibility of cross-study gene-based tests, where Z-statistics from the same markers are combined across previously published GWAS to reap power gains. A shortcoming of our summary statistic based test is that if the estimated correlation structure used in the test is not reflective of the underlying population, the test may suffer from inflated type 1 error. We therefore proposed an modification of the test by adjusting the estimated correlation matrix, which, in general, should help control the error rate. If there is insufficient justification for why the estimated correlation matrix is representative of the underlying population, the test should not be used even with correlation matrix adjustment. If one has the original SNP data, one can perform the dimension-reduced score test proposed in this paper, reducing the dimension to the number of eigenvectors that explains some pre-determined proportion of variation in the data.

Both of the proposed gene-based tests in this paper fail to describe the direction of association between the gene and outcome, instead describing only significance of association. Direction of association is a difficult concept to interpret when a gene is composed of multiple SNPs, with some alleles protective and others a risk factor for the outcome. One goal in gene-based testing might be to gain an understanding of such a concept. Additionally and with regard to dimension reduction approaches, if alleles in a dimension-reduced block of SNPs are both protective and harmful, there could be a loss of power using a dimension-reduction gene-based test. A test that used a priori analyses to decide whether alleles are protective or harmful and, in turn, used that information to inform the dimension reduction process might be another valuable area of research in gene-based testing.

## Abbreviations

OR: Odds Ratio; COPD: Chronic Obstructive Pulmonary Disorder; LD: Linkage Disequilibrium; SNP: Single Nucleotide Polymorphism.

## Competing interests

The authors declare that they have no competing interests.

## Authors’ contributions

DMS developed testing methods, provided the permutation test description, wrote simulations, and analyzed the data. DB provided numerous edits, clearer wording, and biological context for GWAS analysis. TAC, KUL, and EM provided the sequence data for analysis. CL contributed to the Discussion, provided references and edits, contributed knowledge of GWAS to make manuscript more relevant, and gave guidance on tests and dimension reduction. All authors read and approved the final manuscript.

## Supplementary Material

Additional file 1 **R script for test implementation and checking power variation for a permutation-based test.** An R script implementing the summary statistic test with correlation matrix modification can be found at http://db.tt/bZz59KNO. Additionally, the script includes a function with which researchers can see the variation in power of a permutation-based gene-test as a function of placement of the causal marker for a correlation matrix of their choosing. It allows the researcher to see if there would be significant variation so that previously implemented permutation-based tests can either be re-performed using alternative methods or more confidence can be placed in already implemented tests. The code is commented with a description of function arguments and intended use. Click here for file
